# Nonsyndromic Hypodontia Including Bilateral Agenesis of Permanent Maxillary Canines: A Case Report

**DOI:** 10.1155/crid/9300041

**Published:** 2026-08-26

**Authors:** Aiko Kurosaka, Hideto Saijo

**Affiliations:** ^1^ Department of Oral and Maxillofacial Surgery, Kagoshima University Hospital, Kagoshima City, Kagoshima, Japan, kagoshima-u.ac.jp

**Keywords:** case report, cone-beam computed tomography, dental agenesis, hypodontia, maxillary canine

## Abstract

**Background:**

Permanent maxillary canine agenesis is uncommon, and bilateral absence is particularly rare. When eruption is delayed in the canine region, developmental absence must be distinguished from impaction or ectopic development because the diagnosis changes orthodontic and restorative planning.

**Case Presentation:**

A 13‐year‐old Japanese boy was referred because his permanent teeth had not erupted in the maxillary anterior region. Panoramic radiography showed absence of the permanent tooth germs at FDI 12, 13, 23, and 45. Targeted cone‐beam computed tomography confirmed that no impacted or ectopically positioned tooth structures were present at these sites. No ectodermal or craniofacial findings suggested a syndrome. The patient had an Angle Class II first‐molar relationship, with 1‐mm overjet measured at the maxillary right central incisor and 1 mm overbite. A canine relationship could not be assessed because both permanent maxillary canines were absent. The mother had agenesis of all four permanent second premolars, the sister had bilateral agenesis of the mandibular second premolars, and the father had no dental agenesis. A lateral cephalogram and complete orthodontic records were not available during the diagnostic assessment.

**Conclusion:**

This case represents nonsyndromic moderate hypodontia with an unusual bilateral maxillary canine pattern and variable expression of tooth agenesis within the family. Clinical examination, panoramic imaging, and problem‐oriented cone‐beam computed tomography established the diagnosis and supported the need for staged multidisciplinary follow‐up during growth.

## 1. Introduction

Tooth agenesis is the developmental failure of one or more teeth to form. Excluding third molars, mandibular second premolars and maxillary lateral incisors are among the teeth most often affected [1, 2]. The clinical burden varies with the number and position of the missing teeth, and a four‐tooth pattern has been categorized as moderate hypodontia in clinical studies [3]. Tooth agenesis may occur as an isolated finding or as part of a syndrome; therefore, the dental pattern should be interpreted together with the medical history and extraoral examination [2].

Permanent maxillary canines are comparatively stable in development, making their congenital absence unusual. Published case reports and reviews indicate that bilateral maxillary canine agenesis is especially rare and may coexist with agenesis at other sites [4–6]. This distinction matters clinically because an unerupted canine is far more commonly impacted or ectopic than developmentally absent. A diagnosis of agenesis leads to different decisions concerning preservation of primary teeth, space closure or opening, and the timing of definitive tooth replacement.

We report a 13‐year‐old boy with bilateral agenesis of the permanent maxillary canines accompanied by agenesis of the maxillary right lateral incisor and mandibular right second premolar. The case is also notable for hypodontia with a different distribution in the patient′s mother and sister. The purpose of this report is to describe the diagnostic reasoning, the role of imaging, the occlusal findings that were available, and the considerations that will guide future multidisciplinary care.

## 2. Case Presentation

### 2.1. Patient Information and Clinical Findings

A 13‐year‐old Japanese boy was referred to the Department of Oral and Maxillofacial Surgery for evaluation of delayed or absent eruption in the maxillary anterior region. His medical history was unremarkable. There was no history of maxillofacial trauma, extraction of the teeth in question, or a systemic disorder known to interfere with dental development. General growth and development appeared normal. Examination did not identify abnormalities of the hair, nails, skin, or sweating, and no craniofacial anomaly was observed.

The first‐molar relationship was Angle Class II. Overjet measured at the maxillary right central incisor was 1 mm, and overbite was 1 mm. Because both permanent maxillary canines were congenitally absent, a conventional canine relationship and canine guidance could not be evaluated. A lateral cephalogram, dental casts or digital models, and a complete orthodontic photographic series were not available at the time of this radiology‐focused referral. Consequently, a sagittal skeletal classification and quantitative assessment of incisor inclination were not made.

### 2.2. Timeline

The sequence of the diagnostic assessment is summarized in Table 1.

**Table 1:** Diagnostic timeline. CBCT, cone‐beam computed tomography; FDI, Fédération Dentaire Internationale tooth notation.

Stage	Clinical information and action
Initial referral	Delayed or absent eruption was noted in the maxillary anterior region.
Clinical assessment	No syndromic features were identified. The first‐molar relationship was Angle Class II; overjet and overbite were each 1 mm. The canine relationship was not assessable.
Panoramic radiography	No permanent tooth germs were visible at FDI 12, 13, 23, or 45.
Targeted CBCT assessment	No impacted or ectopically positioned tooth structures were found at the corresponding sites.
Diagnostic conclusion	Nonsyndromic moderate hypodontia was diagnosed, and complete orthodontic and prosthodontic assessment during growth was considered necessary.

### 2.3. Diagnostic Assessment

**Figure 1:**
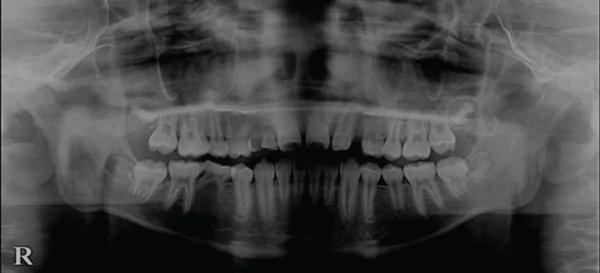
Panoramic radiograph showing absence of the permanent tooth germs at FDI 12, 13, 23, and 45.

Panoramic radiography showed no permanent tooth germs corresponding to the maxillary right lateral incisor, both maxillary canines, and the mandibular right second premolar (FDI 12, 13, 23, and 45, respectively) (Figure 1). The remaining permanent teeth demonstrated development and positioning appropriate to the available radiographic record.

**Figure 2:**
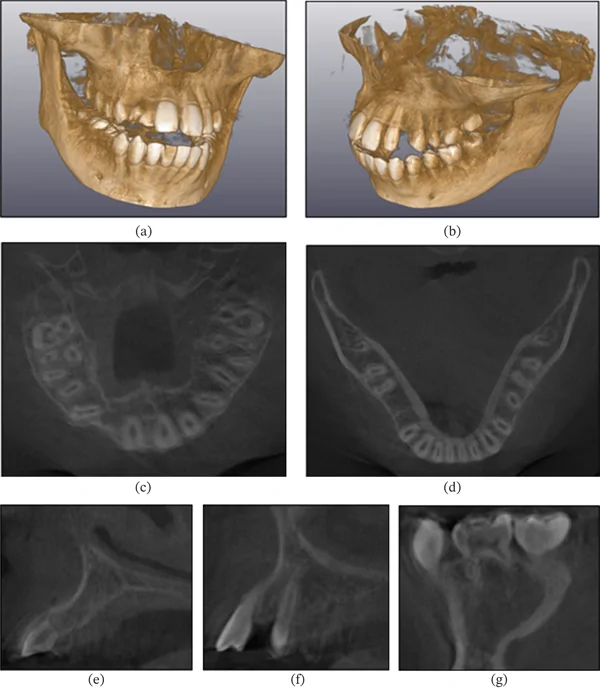
CBCT images. (a, b) Three‐dimensional reconstructions. (c, e) Right maxilla demonstrating absence of tooth germs for FDI 12 and 13. (f) Left maxilla demonstrating absence of the tooth germ for FDI 23. (d, g) Right mandible demonstrating absence of the tooth germ for FDI 45. No impacted or ectopically positioned teeth are visible in the corresponding regions.

The principal differential diagnosis was deep impaction or ectopic development of one or more teeth that could not be localized confidently on the panoramic image. Cone‐beam computed tomography (CBCT) was therefore used to answer this specific diagnostic question. Review of the axial, coronal, and three‐dimensional reconstructions demonstrated no tooth structures in the corresponding regions (Figure 2). Together with the negative extraction history, these findings supported developmental agenesis rather than impaction, ectopia, or acquired absence. Agenesis of four permanent teeth established a diagnosis of moderate hypodontia [3]. Because the clinical examination did not suggest a recognized syndrome, the phenotype was classified as nonsyndromic.

Available panoramic radiographs of first‐degree relatives were also reviewed. The mother had bilateral agenesis of the maxillary and mandibular second premolars, whereas her permanent maxillary canines and lateral incisors were present. The father had a complete permanent dentition without dental agenesis. The patient′s 10‐year‐old sister had bilateral agenesis of the mandibular second premolars. The variation in affected tooth types within the family was documented without assigning a specific inheritance pattern.

### 2.4. Management and Follow‐Up

This report covers the diagnostic phase. No active orthodontic or prosthodontic treatment had been completed during the period described, and longitudinal treatment outcomes were therefore unavailable. The imaging findings indicate the need for comprehensive orthodontic and prosthodontic assessment, including records that were not obtained at the initial referral, before irreversible decisions are made regarding primary‐tooth extraction, space closure, space opening, or definitive tooth replacement. A formal patient or guardian perspective was not recorded for this diagnostic case report.

## 3. Discussion

The present patient had agenesis of four permanent teeth, including both maxillary canines. Although the maxillary right lateral incisor and mandibular right second premolar belong to tooth groups that are commonly affected by agenesis, congenital absence of a permanent maxillary canine is rare, and bilateral absence is rarer still [1, 4–6]. The prevalence of maxillary canine agenesis has been reported in the range of approximately 0.07%–0.13% in the literature summarized by Kambalimath et al. [4]. The combination observed here therefore represents an unusual distribution within otherwise moderate hypodontia.

The family findings support the possibility of a shared predisposition but do not establish a mode of inheritance. Nonsyndromic tooth agenesis is etiologically heterogeneous and may reflect variants in several developmental pathways as well as modifying environmental factors [2, 7]. In this family, the mother, proband, and sister had different missing‐tooth patterns, whereas the father was unaffected. Without genetic testing or a larger pedigree, it would be inappropriate to infer a single‐gene mechanism or a Mendelian transmission pattern. The family history is nevertheless clinically relevant because it may prompt earlier dental surveillance of relatives.

The diagnostic sequence also illustrates a measured use of three‐dimensional imaging. Panoramic radiography provided the initial overview and suggested agenesis. CBCT should not be obtained routinely whenever a tooth is unerupted; however, it can be useful when two‐dimensional imaging does not adequately resolve whether a canine is deeply impacted, ectopic, or associated with adjacent structures [8]. In this case, CBCT addressed that focused differential diagnosis and prevented management based on a mistaken assumption of impaction. After agenesis has been established, further radiography should be individualized to clinically relevant questions and kept as limited as reasonably achievable.

The available occlusal findings must also be interpreted within the limits of the records. The patient had an Angle Class II first‐molar relationship with 1‐mm overjet and 1‐mm overbite. These dental observations do not by themselves define the sagittal skeletal relationship. Moreover, the absence of both permanent maxillary canines made conventional assessment of canine relationship and canine guidance impossible. Because no lateral cephalogram was available, neither skeletal Class I versus Class III nor bimaxillary proclination could be confirmed or excluded. Definitive orthodontic diagnosis will require cephalometric analysis, facial assessment, arch‐length evaluation, and study models or digital scans.

Preservation of a healthy primary tooth can sometimes maintain function, arch length, and alveolar bone while a child is growing. Evidence concerning retained primary second molars in patients with premolar agenesis shows that some teeth remain serviceable, but their prognosis varies [9]. Decisions should therefore be tooth‐specific and should consider root resorption, ankylosis or infraocclusion, caries, periodontal health, crown morphology, and the intended direction of orthodontic movement. In the present patient, extraction of a retained primary tooth should not be prescribed solely because the permanent successor is absent; the timing should follow a complete multidisciplinary plan.

Space management is particularly complex because the patient lacks both maxillary permanent canines as well as the maxillary right lateral incisor. Conventional canine substitution for an isolated missing lateral incisor is not directly applicable when the canines themselves are absent. Potential strategies include maintaining or opening selected spaces for prosthetic replacement, closing spaces through premolar substitution with reshaping where appropriate, or using a combined approach across the two sides of the arch. Studies of isolated maxillary lateral incisor agenesis describe esthetic, periodontal, and functional trade‐offs between orthodontic space closure and prosthetic replacement [10–12]. Those data can inform discussion with the family, but they cannot be transferred uncritically to this uncommon four‐tooth pattern.

Management should therefore be staged. During growth, clinically healthy primary teeth may be monitored, and an interim resin‐bonded restoration may be considered if an esthetic space requires replacement. Definitive implant‐supported rehabilitation is generally delayed until craniofacial growth is complete to reduce the risk of progressive infraocclusion and positional discrepancy [13]. Temporary anchorage devices may provide additional anchorage for space redistribution or implant‐site development in selected patients [14]. The eventual plan must incorporate the facial profile, skeletal pattern, available bone, arch discrepancy, periodontal tissues, growth status, and the patient′s preferences; these factors cannot be resolved from the current diagnostic records alone.

The main strengths of this report are documentation of a rare bilateral canine phenotype, review of first‐degree family radiographs, and confirmation that no impacted or ectopic tooth structures were present. The limitations are the absence of a lateral cephalogram and complete orthodontic records, lack of genetic testing, absence of longitudinal treatment and outcome data, and lack of a formally recorded patient perspective. These limitations have been made explicit so that the diagnostic findings are not overextended into a definitive orthodontic treatment prescription.

## 4. Conclusion

A 13‐year‐old boy was diagnosed with nonsyndromic moderate hypodontia involving bilateral permanent maxillary canine agenesis together with agenesis of FDI 12 and 45. Clinical examination, panoramic radiography, and problem‐oriented CBCT differentiated developmental absence from impaction or ectopic development. The variable hypodontia pattern in the mother and sister suggests familial susceptibility without defining an inheritance mechanism. Complete orthodontic records and staged multidisciplinary planning are required before decisions are made about primary teeth, space management, interim restoration, or definitive implant treatment.

## Author Contributions

Aiko Kurosaka: conceptualization, data curation, investigation, visualization, writing—original draft, and writing—review and editing. Hideto Saijo: conceptualization, supervision, validation, and writing—review and editing.

## Funding

No funding was received for this manuscript.

## Disclosure

All authors have read and approved the final version of the manuscript. Aiko Kurosaka, the corresponding author and manuscript guarantor, had full access to all of the data in this study and takes complete responsibility for the integrity of the data and the accuracy of the data analysis.

## Ethics Statement

Formal ethical approval was not required for this single anonymized case report. Written informed consent for publication was obtained, as detailed below. This case report was prepared in accordance with the CARE guidelines [15].

## Consent

Written informed consent for publication of the clinical information and images was obtained from the patient and the patient′s legal guardian. Written consent was also obtained for the use of anonymized family dental histories and radiographs.

## Conflicts of Interest

The authors declare no conflicts of interest.

## Data Availability

Data sharing is not applicable to this article as no datasets were generated or analyzed during the current study.
